# Altered Presence of Cancer Stem Cell ALDH1/2 in Oral Leukoplakias and Squamous Cell Carcinomas

**DOI:** 10.7759/cureus.40836

**Published:** 2023-06-22

**Authors:** Vasileios Zisis, Konstantinos Paraskeuopoulos, Athanasios Poulopoulos, Prashanth Panta, Dimitrios Andreadis

**Affiliations:** 1 Oral Medicine/ Pathology, Aristotle University of Thessaloniki, Thessaloniki, GRC; 2 Oral & Maxillofacial Surgery, Aristotle University of Thessaloniki, Thessaloniki, GRC; 3 Oral Medicine and Radiology, Malla Reddy Institute of Dental Sciences, Hyderabad, IND

**Keywords:** oscc, aldh, oral leukoplakia, oral cancers, cancer stem cells

## Abstract

Introduction: Cancer stem cells (CSCs) are responsible for initiating the process of carcinogenesis by enabling the self-renewal and self-proliferation of the cancer cells. This study aimed to investigate the presence of epithelial cells with cancer stem cells characteristics (ALDH+) in the early stages of oral precancerous lesions (Oral Leukoplakias) and the frequency of these cells in the different stages of oral squamous cell carcinomas (OSCCs).

Materials & methods: The aim of this study was the detection of the immunohistochemical pattern of expression of CSC protein-biomarker ALDH1&2 (sc-166362, Santa Cruz Co, Dallas, Texas, USA) in paraffin-embedded samples of 30 cases of leukoplakia of all degrees of dysplasia and 21 cases of oral squamous cell carcinomas (OSCC) of all degrees of differentiation compared to the histologically normal oral epithelium. The samples were retrieved from 2009-2019 from the archives of the Department of Oral Medicine/Pathology, School of Dentistry, Aristotle University of Thessaloniki, Greece. The samples were evaluated through a three-tier scale (positive cells Ι: 6-35%, ΙΙ: 36-70%, ΙΙΙ: 71-100%). Statistical analysis was performed through SPSS Pearson Chi-square, and the significance level was set at 0.05 (p=0.05).

Results: The staining of ALDH1&2 was observed mildly in the cell membrane of cells in the stratum spinosum of the normal epithelium and the cell membrane of cells in the stratum basale of the normal epithelium, characteristically at the interface point with the basal membrane. ALDH1&2 were expressed significantly more in the OSCC than in the leukoplakia (p-value=0.0001) and the normal epithelium (p-value=0.0001). Mainly, ALDH1&2 were expressed significantly more in the severely and moderately dysplastic oral leukoplakia compared to the mildly dysplastic and non-dysplastic leukoplakia (p-value=0.001).

Discussion: The characteristic expression of ALDH in potentially malignant oral and OSCC lesions suggests the presence of CSCs and their possible implication in the early stages of oral tumorigenesis, even at the stage of oral leukoplakia.

## Introduction

Leukoplakia

The term "oral potentially malignant disorder (OPMD)" is attributed to oral mucosal disorders/lesions, which exhibit an increased risk for malignant transformation compared to healthy mucosa [1]. The most common OPMD is the Oral Leukoplakia (OL) [2]. Leukoplakia is conservatively divided into non-dysplastic leukoplakia, mildly dysplastic leukoplakia, moderately dysplastic leukoplakia, and severely dysplastic leukoplakia, depending on the degree of dysplasia noticed on the histological level [3]. Otherwise, leukoplakia may be divided into two groups based on the binary taxonomy proposed by the WHO, 2005 [4]. The first group includes non-dysplastic leukoplakia and mildly dysplastic leukoplakia, whereas the second group includes moderately dysplastic leukoplakia and severely dysplastic leukoplakia (binary taxonomy) (WHO, 2005) [5,6]. Dysplasia is described as a collection of epithelial cell histological processes that indicate phenotypic deviation. The more the coexistence of histological processes, the greater the severity of dysplasia [7,8].

Oral squamous cell carcinoma

Oral squamous cell carcinoma arises from cells of the stratified squamous epithelium. Its development location influences biological behavior, prognosis, and therapeutic therapy [9]. The oral squamous cell carcinoma is characterized according to its histological appearance. Specifically, differentiation is divided into three categories: high, medium, and low. Well-differentiated carcinoma consists of dermis-infiltrating strands of neoplastic cells with a detectable propensity toward keratinization (keratin spheres). The low-differentiated end consists of cells that resemble primordial undifferentiated cells with a high degree of variety (WHO Classification, 2017) [10]. Approximately 49.5% of recorded squamous cell carcinomas are of good differentiation, 34.3% of moderate differentiation, and 16.2% are of low differentiation [11]. 

Cancer stem cell biomarkers ALDH1&2

Cancer stem cell biomarkers are used to detect subpopulations of cancer stem cells and are separated into two different subgroups: markers of embryonic stem cells and markers showing the presence of stem cell traits in certain cells (Stemness). These indicators relate to the molecular processes and mechanisms that control the traits of cancer stem cells and are essential to their survival. Aldehyde dehydrogenases are a group of enzymes that catalyze the oxidation of aldehydes and function as Stemness markers. They convert aldehydes to carboxylic acids [12]. In addition, it mediates the oxidation of retinol to retinoic acid, which regulates cell differentiation [13]. The main structural features of ALDH are the cofactor binding domain, the active site, and the oligomerization mechanism. The structural difference between ALDH1-ALDH 2 lies in a translocation of 5Å of the nicotinamide portion of the cofactor's binding area [14]. Aldehyde dehydrogenase is increasingly employed as a biomarker of cancer stem cells in squamous cell carcinoma since cells positive for it exhibits phenotypic flexibility, generate typical cancer cell spheres and islets in vitro, and exhibit self-renewal [15]. Aldehyde Dehydrogenase increases the likelihood of cancer recurrence and is associated with the development of resistance to both chemotherapy and radiotherapy [16]. ALDH1 has been identified in the cytoplasm of the squamous cell epithelium of the mouth [17].

The hypothesis and objectives of this study

The purpose of the current research was to examine the immunohistochemical model of the expression of Aldehyde Dehydrogenase 1&2 (Aldehyde dehydrogenase 1&2-ALDH 1&2) in cases of oral leukoplakia of all dysplastic degrees and cases of oral squamous epithelium carcinoma of all differentiation degrees. An important study topic was whether ALDH-positive cells are found at the earliest stages of dysplasia and carcinogenesis. The corresponding study hypothesis to be accepted or denied was if a positive correlation exists between the degree of dysplasia of oral leukoplakia, the degree of differentiation of oral squamous cell carcinoma, and the quantitative expression of ALDH in the respective cell types. The whole research portion of the study adhered to international and national research methodological standards and was authorized by the Ethics Committee of the Department of Dentistry at its meeting on 03/07/2019, with Protocol Number 8/03.07.2019.

## Materials and methods

This study examines the immunohistochemical expression of the Anti-ALDH1-2 index (sc-166362, Santa Cruz Biotechnology, Dallas, Texas, USA) in tissue samples from cases of oral leukoplakia of all degrees of dysplasia and oral squamous cell carcinomas of all degrees of differentiation in comparison to cases of normal mucosa (neighboring healthy epithelium from reactive hyperplasias e.g., fibroma). The full laboratory process was conducted at the Laboratory of Stomatology, Dental School, Aristotle University of Thessaloniki. In particular, the paraffin-embedded tissue samples belonged to patients with leukoplakia or cancer who visited the Laboratory of Stomatology, Dental School, Aristotle University of Thessaloniki (A.U.Th.) between 2009 and 2019, and a biopsy was conducted as part of the normal diagnostic procedure.

These samples were put in formaldehyde (10%) immediately after surgical removal and later preserved in paraffin. The inclusion criteria were the presence of enough biological material in the paraffin cubes for the immunohistochemistry staining with Anti-ALDH1&2, the highest feasible equitable involvement of men and women of comparable ages, behaviors (smoking-alcohol consumption), and medical history that did not influence the development of the biomarker. The exclusion criterion was insufficient biological material in the paraffin cubes. In detail, 30 OL cases were chosen and further divided into two subgroups according to the WHO 2005 binary taxonomy for oral leukoplakia. The first subgroup included 14 cases of nondysplastic and mildly dysplastic leukoplakia, whereas the second included 16 cases of moderately and severely dysplastic leukoplakia. In detail, 21 cases of oral squamous cell carcinoma were chosen, according to the WHO 2017 OSCC taxonomy, based on the degree of differentiation, which were further divided into two subgroups. The first subgroup included 16 cases of moderately and poorly differentiated OSCC, whereas the second subgroup included 5 cases of well-differentiated OSCC. The control group comprised 5 cases of normal oral epithelium adjacent to reactive hyperplasias (fibroma). After collecting the histology samples, the tissue was fixed in a 10% formaldehyde solution to preserve its cellular structure, and it was then embedded in paraffin to harden the tissues for long-term preservation and enable the cutting of fine incisions. Using a Jung Biocut 2035 microtome, 4m-thick incisions were made based on the original hematoxyline-stained incision (Leica, Germany). For the immunohistochemical technique application, the incisions were mounted on slides (Polysine Superfrost Plus, Thermo scientific Menzel Gläser, Braunschweig, Germany), and the associated hematoxyline incisions were examined for comparison. The following protocol was followed when the material was further processed using the immunohistochemical technique with the Anti-ALDH1&2 antibody (sc-166362, Santa Cruz Biotechnology, USA) at a dilution of 1:100 using the Dako Envision System Flex + secondary stain detection system (Dako Denmark A/S). Specifically, staining was achieved with the Anti-ALDH1&2 antibody during the following steps of the immunohistochemistry (IHC) technique and included Antigen recovery (Antigen Retrieval), Application of primary antibody, Utilization of the Dako Envision System's secondary stain detection system, Chromogenic agent application (Dako Dab Envision Denmark A/S) (Chromogen), and employment of hematoxyline (Hematoxylin). The incision was then affixed to the mounting plate and coated to protect and preserve the preparation over time. The immunohistochemical staining was evaluated by microscopically examining the incisions to observe and record the results. For microscopy and recording of results, an OLYMPUS CX31 microscope (OLUMPUS LS, Japan) and an OLYMPUS SC30 camera were utilized (OLYMPUS SOFT IMAGING SOLUTIONS, Muenster, Germany).

The cases were divided into three categories: Leukoplakia (B), Cancer (C), and Normal (D). Leukoplakia (B) and Cancer (C) were further subdivided into two subcategories, respectively: Moderately and severely dysplastic Leukoplakia (B1), Mildly dysplastic and nondysplastic Leukoplakia (B2), Moderately and poorly differentiated OSCC (C1) and Well-differentiated OSCC (C2). The samples were evaluated through a three-tier scale (positive cells Ι: 6-35%, ΙΙ: 36-70%, ΙΙΙ: 71-100%) (Table 1).

**Table 1:** Quantitative evaluation of the staining

ALDH+ cells	
0-5%	0
6-35%	I
36-70%	II
>71%	III

Negative staining was defined as the presence of less than 5% of positively stained cells. In detail, the three-tier scale was defined as follows: Ι: Presence of positive cells in one-third of the epithelium, ΙΙ: Presence of positive cells in two-thirds of the epithelium, ΙΙΙ: Presence of positive cells throughout the epithelium. The immunohistochemical staining was evaluated as positive when the cytoplasm was depicted in brown. Depending on the degree of positivity of ALDH 1&2 (6-35%, 36-70%, 71-100%), each case of categories B, C & D and subcategories B1, B2, C1, C2 acquired a value of 1 to 3 [18]. Statistical analysis was performed through the SPSS software (IBM Corp. Released 2017. IBM SPSS Statistics for Windows, Version 25.0. Armonk, NY: IBM Corp.) with Pearson Chi-square test and the Fisher's Exact test depending on the sample size and the significance level was set at 0.05 (p=0.05).

## Results

**Figure 1:**
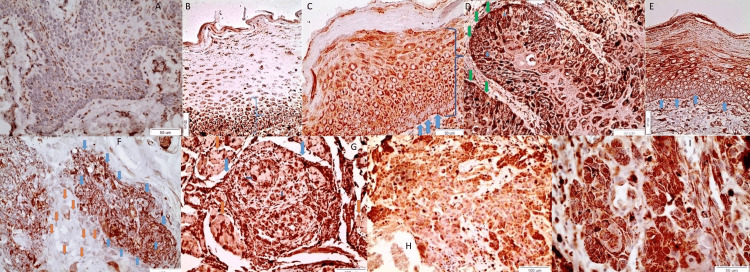
ALDH pattern of expression in normal oral epithelium, leukoplakia, and cancer

In the normal oral epithelium (D), it was found that the ALDH 1&2 is mildly expressed in the cell membrane of individual cells, mainly of the spindle layer but also cells of the basal layer characteristically at the interface point with the basal membrane (Figure 1A, x40) whereas perinuclear positivity is also observed. Respectively, in cases of nondysplastic and mildly dysplastic leukoplakia, significantly more positive expression was observed, which was more strongly detected in the lower 1/3 of the epithelium and, in particular, in the cell membrane and the cytoplasm of epithelial cells (Figure 1B, x40). In contrast, in moderately and severely dysplastic leukoplakia, positive expression of ALDH 1&2 was observed in more than 2/3 of the epithelium (Figure 1C, x40). In particular, strong staining was observed almost throughout the entire epithelium except for its upper layer (keratin). The staining is cytoplasmic and membrane but not nuclear. In all cases of leukoplakia, a characteristic positive expression of ALDH1&2 was observed in the interface area of the cells of the basal layer of the epithelial cells with the basal membrane. In the overlying, severely dysplastic epithelium covering a neighboring well-differentiated OSCC, a membrane and cytoplasmic expression of ALDH1&2 is observed. Especially in the area where the infiltration of neoplastic cells begins, the expression of ALDH1&2 in the interface area of these cells with the basal layer (arrow) is lost (Figure 1D, x40). Nuclear staining is also observed in individual cells. A similar model of the expression of ALDH1&2 is also noticed in the severely dysplastic epithelium neighboring both moderately and poorly differentiated cancerous foci.

Diffuse cytoplasmic staining of the spindle layer and membrane staining of the basal and parabasal layers are noticed (Figure 1E, x40). Cancerous focus with masses of small cells strongly positive to ALDH1&2 (blue arrows) is noticed. On the contrary, cancer cells of larger size remain negative to ALDH1&2 (orange arrows) in moderately dysplastic OSCC (Figure 1F, x20). Cancerous focus (blue arrows) between muscle bundles (orange arrows) in moderately differentiated OSCC (Figure 1G, x20). Positive nuclear staining is also observed in individual cells (purple arrows). Cancerous foci with scattered cancer cells are observed in poorly differentiated OSCC (Figure 1H, x20) (Figure 1I, x40). The aforementioned observations are summarised in Table 2.

**Table 2:** Summary of the descriptive results from the immunohistochemical images. OSCCs: oral squamous cell carcinomas

Figure	Condition	Location
Figure 1A	Normal oral epithelium	Basal and spindle cell layers
Figure 1B	Mildly dysplastic and non-dysplastic leukoplakia	Lower 1/3 of the epithelium
Figure 1C	Severely and moderately dysplastic leukoplakia	Lower 2/3 of the epithelium
Figure 1D	Infiltration of neoplastic cells initiating OSCC	Interface area between basal cell layer and basal membrane
Figure 1E	Severely dysplastic epithelium neighboring cancerous foci	Lower 2/3 of the epithelium
Figure 1F	Cancerous focus	ALDH positive and negative cells
Figure 1G	Cancerous focus	Surrounded by muscle bundles
Figure 1H	Cancerous focus	Scattered cells
Figure 1I	Cancerous focus	Scattered cells

From the statistical analysis among the categories Leukoplakia, Cancer, and Normal oral epithelium and the subcategories leukoplakia moderately and severely dysplastic, leukoplakia nondysplastic and mildly dysplastic, moderately and severely differentiated OSCC, and well-differentiated OSCC, emerged the following observations. ALDH1&2 was expressed significantly more in the OSCC compared to the Leukoplakia (Pearson Chi-square test, p value= 0.001) and the normal epithelium (Pearson Chi-square test, p value= 0.001), respectively (Table 3). Particularly, ALDH1&2 was expressed significantly more in the severely and moderately dysplastic leukoplakia compared to the mildly dysplastic and non-dysplastic leukoplakia (Pearson Chi-square test, p value= 0.001) (Table 3).

**Table 3:** Summary of the statistically significant results.

Higher expression	Lower expression	P value
OSCC	Leukoplakia	Pearson Chi-square test, p value= 0.001
OSCC	Normal oral epithelium	Pearson Chi-square test, p value= 0.001
Severely and moderately dysplastic leukoplakia	Mildly dysplastic and non-dysplastic leukoplakia	Pearson Chi-square test, p value= 0.001

However, the expression of ALDH1&2 was not statistically significantly altered between the moderately and severely dysplastic leukoplakias and the well-differentiated OSCCs (Fischer's exact test, p-value =0.670). However, the expression of ALDH1&2 was not statistically significantly altered between the moderately and severely dysplastic leukoplakias and the poorly and moderately differentiated OSCCs (Fischer's exact test, p-value =0.240). However, the expression of ALDH1&2 was not statistically significantly altered between the poorly and moderately differentiated OSCCs and the well-differentiated OSCCs (Fischer's exact test, p-value =0.115). The aforementioned findings imply that the expression of aldehyde dehydrogenase is detected at higher levels in general in cancer. In particular, higher levels are detected in poorly and moderately differentiated cancer, in well-differentiated cancer, and in moderately and severely dysplastic leukoplakia, while lower levels are detected in mildly and nondysplastic leukoplakia and the normal oral epithelium (Table 4).

**Table 4:** Summary of the results.

	ALDH1&2 levels
Poorly and moderately differentiated cancer	++
Well-differentiated cancer	++
Moderately and severely dysplastic leukoplakia	++
Mildly and non-dysplastic leukoplakia	+
Normal oral epithelium	+

Both cytoplasmic and membrane staining of the epithelial cells of the basal, parabasal, and spindle layers were observed. Nuclear staining of individual cells was noticed in OSCCs. Positive staining of cancer stem cells and absence of staining of cancer cells in cancerous foci were observed.

## Discussion

Cancer stem cells are a tiny fraction of self-renewing cells detected inside malignant tumors. Their clinical relevance stems from the fact that they are now believed to be responsible for forming metastases and the evolution of chemotherapy and radiation resistance. Their discovery and identification are deemed significant since they will be for the development of more focused medicines that will prolong patients' life expectancy and enhance their quality of life [19]. In the oral cavity, cancer stem cells are detected both into cancerous lesions and potentially malignant lesions [20]. According to the study hypothesis, there is a positive correlation between the degree to which cancer stem cells are found, the degree of dysplasia of OPMDs, and the degree of differentiation of OSCC. The literature results regarding ALDH expression are summarised in Table 5.

**Table 5:** Literature results regarding ALDH expression.

ALDH subtypes	Literature results
ALDH1	Positive expression of ALDH1 in oral leukoplakia is associated with a threefold increased risk of malignant transformation in OSCC, suggesting that ALDH1 promotes the transformation process [21].
	ALDH1 is identified more often in OSCC compared to normal mucosa and mild dysplasia [22]. It is expressed throughout the epithelium except for the keratin layer [22].
ALDH1A1	ALDH1A1 is more prevalent in leukoplakia than in OSCC [23]​​​​​​.
	ALDH1A1 is present in all leukoplakia cases and is likely linked with a significant probability of malignant transformation, particularly in the basal and parabasal layers [23].
	In OSCC cases, scattered cells positive for ALDH1A1 and the lack of staining of keratin pearls are observed [22].
	In addition, ALDH1A1 is overexpressed in leukoplakia cases and underexpressed in OSCC cases [​21], serving as a marker for future cancer cells [​24].
ALDH2	ALDH 2 is overexpressed in OSCC of every degree of differentiation [23].
	ALDH 2 is normally expressed in the context of cell respiration, regardless of alcohol use. In the event of chronic alcohol abuse, however, the overexpression of ALDH 2 is expected since the oral mucosa is exposed to carcinogens, and its regeneration requires 14 days [​25].

Therefore, it is documented that the overall expression of ALDH1&2 increases as the degree of dysplasia increases and the malignant transformation process commences. We examined ALDH 1&2 and discovered the enzyme in nearly all our tissue samples. The qualitative (intensity of staining) but primarily quantitative (extent of the involved epithelium) presence of ALDH is related to the degree of dysplasia of leukoplakia and the degree of cancer differentiation. The characteristic cytoplasmic staining of ALDH in leukoplakia and cancer was observed, which is also reported by other authors [16,22,24,25,26], but nuclear and membrane staining was also observed, which has not been recorded or reported by other authors as possible staining in cancer stem cells in OSCC. In contrast, membrane expression of ALDH1 has been observed in kidney cancer [27], whereas cytoplasmic and membrane expression of ALDH1 is observed in bronchial epithelial cells and type 2 alveolar cells in lung cancer [13]. ALDH1A1 is expressed in colorectal carcinomas, and its nuclear expression indicates a favorable prognosis [28,29]. Particularly, a pattern of expression of ALDH was frequently observed in the area of the interface between the basal cells of the oral epithelium and the basal membrane, leading to the hypothesis that this receptor participates, for example, in the formation of hemidesmosomes or functions as a sign of intense metabolic activity in the basal cell / basal membrane region. Particularly in OSCC cases, there is membrane expression in the cells of the basal and suprabasal layer and cytoplasmic in the overlying spindle layer (the question arises as to whether the cellular metabolism is altered), as well as nuclear staining in individual cells in severely dysplastic epithelium adjacent to OSCC and moderately differentiated OSCCs. Cancer stem cells' positive staining and absence of cancer cells were noticed in cancerous foci beneath the squamous epithelium. No other authors have observed or recorded these expression patterns.

The main novelty of our study is that in addition to comparing leukoplakia and cancer as categories, we also compare all the subcategories of each disease despite the small number of cases (1. leukoplakia nondysplastic and mildly dysplastic, 2. leukoplakia moderately and severely dysplastic, 3. well-differentiated cancer, 4. poorly and moderately differentiated cancer) as well as carefully selected normal oral epithelium cases. No correlations were established between the degree of positivity of ALDH1&2 and the staging of CTNM (clinical TNM), i.e., in terms of the clinical prognosis of patients over time (clinical-pathological correlation), nor with the localization of cancers.

## Conclusions

ALDH1&2 is naturally present in tissues and increase, among other things, due to inflammation, making comparisons with normal cases essential. Particularly, physiological cases are required in each batch of incisions undergoing the immunohistochemical technique to prevent the creation of erroneous data, which will lead to erroneous associations. Future studies in larger samples of patients and in conjunction with other cancer stem cell biomarkers may illustrate even better the crucial role ALDH performed in oral carcinogenesis.
